# Prioritisation of assessments, diagnostic classifications, and outcome measures in Perthes disease: a Delphi survey of international health professionals

**DOI:** 10.1007/s00402-026-06392-3

**Published:** 2026-06-25

**Authors:** Stephanie Ball, Luke M. Davies, Kelly Gray, Verity Pacey

**Affiliations:** Faculty of Medicine, Health and Human Sciences, Macquarie University, Sydney, Australia

**Keywords:** Legg-Calve-Perthes disease, child, orthopaedic

## Abstract

**Introduction:**

The aim of this study is to reach consensus and prioritise the most clinically important assessments, diagnostic classifications, and outcome measures by an international panel of experts for Legg-Calve-Perthes disease.

**Materials and methods:**

A three-round modified e-Delphi survey was conducted between April and May 2025. Participants rated the clinical importance of 14 clinical assessments, 26 radiological assessments, eight diagnostic classifications, and 26 outcome measures identified by a scoping review. Consensus was defined as ≥ 75% agreement. In the final round, items in each category were prioritised for overall condition and by specific age groups (< 6 years, 6–8 years, > 8 years).

**Results:**

Thirty-eight health professionals, from nine countries participated in round 1. Thirty-four completed all three rounds, a retention rate of 89.5%. Consensus and prioritisation for clinical use were achieved for 10 clinical assessments, three radiological assessments, three diagnostic classifications, and three outcome measures.

**Conclusion:**

Paediatric specialists have reached consensus and prioritised methods to evaluate and diagnose Legg-Calve-Perthes disease in clinical practice. These results represent an important step towards improving continuity of care and the foundation for future development of clinical practice guidelines.

**Supplementary Information:**

The online version contains supplementary material available at 10.1007/s00402-026-06392-3.

## Introduction

Legg-Calve-Perthes disease (Perthes disease) is a rare self-limiting avascular necrosis (AVN) of the developing proximal capital femoral epiphysis, the pathophysiology of which remains unknown [17]. The secondary effects of Perthes disease include pain, hip joint deformity, and functional limitations, all of which impact physical activity, participation, and quality of life in children and adolescents [12, 17]. Worldwide prevalence of Perthes disease is 0.4–29.0 per 100,000 children and adolescents [20]. Perthes disease is more common in boys than girls (ratio 5:1), with peak incidence occurring between 4 and 7 years of age, while later onset (age > 8 years) is associated with greater disease severity and variability in long-term outcomes [15, 26, 28]. Management approaches vary according to disease stage, age at onset, physician preference, and regional practice patterns; however, the primary aim remains to maintain or optimise hip congruency and promote spherical growth of the femoral head [5, 17]. Numerous approaches to assess, diagnose, and evaluate outcomes in Perthes disease have been reported within the literature, resulting in substantial heterogeneity across clinical and research practice [2]. This variability limits comparability between studies, complicates interpretation of management outcomes, and may contribute to inconsistent clinical decision-making and patient care.

Currently, no standardised approaches exist for assessing, diagnosing or evaluating outcomes in children and adolescents with Perthes disease in either research or clinical practice [2, 19]. A core outcome set for Perthes disease has been developed to improve consistency in outcome reporting and communication between health professionals and families [19]; however, the specific methods to assess or evaluate these outcomes were not defined [19]. A recent scoping review identified substantial variation in clinical assessments, radiological assessments, diagnostic classifications, and outcome measures used in Perthes disease, with few outcome measures validated for paediatric populations [2]. While radiographs were consistently used for diagnosis, monitoring, and post-operative evaluations, variability in the classification systems used for interpretation further contributed to inconsistency across clinical and research practice [2]. The vast number of measurement tools and classifications in use across health domains presents challenges for both clinical and research settings.

Given the absence of strong literature and no clear standard, achieving expert consensus is needed to improve consistency of reporting in clinical practice and research [25]. Establishing consensus by experts on the most clinically relevant, specific, and reliable methods for assessing, diagnosing, and evaluating outcomes in Perthes disease is necessary to foster improved communication, consistency of care, and comparability across studies and healthcare settings to facilitate improved patient outcomes. Therefore, the aim of this study is to reach consensus and prioritise the most important assessments, diagnostic classifications, and outcome measures by expert health professionals for use in clinical practice for children and adolescents with Perthes disease.

## Methods

### Design

A 3-round modified e-Delphi was conducted between April and May 2025. The Delphi technique is a structured process in which experts provide their opinions on a particular issue in a series of ‘rounds’, each round builds upon the previous one, whilst maintaining panel anonymity aiming to reach consensus [3]. This technique is useful when limited research on a topic exists, when knowledge is uncertain and/or incomplete in a particular area, and when the opinions of industry experts is superior to individual opinion [2]. The Delphi methodology was suitable in this context, due to its electronic and remote nature enabling participation from expert health professionals across diverse clinical settings and geographical regions [3, 25]. This study was guided by the Conducting and REporting DElphi Studies (CREDES) (Online Resource 1) [16]. The research team developed purpose-built surveys using Qualtrics, a cloud-based survey platform (Qualtrics, Provo, UT). Ethics were obtained via the Human Research Ethics Committee at Macquarie University (#18453).

### Participants

International experts in the field of Perthes disease were recruited via the research teams academic, research, and clinical networks, as well as recent authors from relevant studies, and healthcare professionals working within paediatric tertiary facilities who manage children and adolescents with Perthes disease. Through purposeful sampling, potential participants were contacted directly via email through professional networks and publicly available information. Participants who completed all three survey rounds were offered a financial honorarium.

All participants were required to be proficient in English and provide informed consent. Health professionals were eligible if they were currently professionally registered and manage paediatric patients (0–18 years) with Perthes disease within the last three years. Researchers were eligible if they had conducted and published studies on Perthes disease within the last three years. Prior to round 1 survey, participants completed screening and demographic data survey (age, gender, country, profession, years’ experience, workplace setting) questions to ensure eligibly criteria were met. Ineligible participants were excluded from the study. All survey rounds were open for two weeks, with a one-week interval between each round. Non-responders were provided with email reminders at one week, three days, and on the day of the survey deadline. Incomplete surveys or non-responding participants were excluded from subsequent rounds.

### Survey development

The survey was informed by a recent scoping review [2] with modifications undertaken to ensure assessment or diagnostic classifications developed for non-Perthes disease hip conditions were excluded and both parent and child version of outcome measures were included. Round 1 comprised 14 clinical assessments, 26 radiological assessments, eight diagnostic classifications, and 26 outcome measures (21 clinical, five radiological). A participant manual was provided to panel members with a description, age range for use, psychometric properties, and links to diagnostic classifications and outcome measures listed in the survey. Order of items in each category were listed alphabetically, and all participants completed each round individually.

#### Round 1

Panel members were differentiated based on their involvement of diagnosing Perthes disease. Those who diagnose were presented with all four categories–clinical assessments, radiological assessments, diagnostic classifications, and outcome measures–whilst those who don’t diagnose were presented with clinical assessments and outcome measures only, to account for differences in scope of practice in various international settings. Panel members rated items in each category as ‘essential’, ‘important’, or ‘unimportant’ for use in clinical practice with children and adolescents with Perthes disease. A priori for consensus was defined as ≥ 75% of participants rating items as “essential” or “important” [10, 21]. Items not reaching consensus were excluded prior to round 2. Panel members were invited to provide general comments and suggestions for additional items to be added in round 2.

#### Round 2

Panel members were presented with summary of round 1 results, presented as percentage agreement stacked bar graphs, and an updated participant manual with diagnostic classifications and outcome measures linked to core outcome set for Perthes disease [19]. Two researchers (SB, VP) analysed free-text responses and added 16 items to round 2 survey that met the scope of the Delphi survey. Panel members were asked to rate whether they “agree” or “disagree” with each item’s use in clinical practice and whether their responses would change based on different age groups or stages of disease. A priori for consensus was defined as ≥ 75% of participants rating “agree” [10, 21]. Items not reaching consensus were excluded prior to round 3.

#### Round 3

Panel members were presented with summary of round 2 results, presented as percentage agreement stacked bar graphs and an updated participant manual with assessments, diagnostic classifications, and outcome measures linked to the core outcome set for Perthes disease [19]. Following analysis of free-text responses, round 3 asked panel members to prioritise the remaining items in each category for use in clinical practice for Perthes disease ‘overall’ (across any age or stage of Perthes disease), as well as by age groups defined as < 6 years, 6–8 years, and >8years, with ‘one’ representing the highest priority.

### Data analysis

Frequencies and percentages of agreement were calculated for individual items within each category in the first and second rounds. Where the original and modified versions of a tool scored > 75% in round 1, the results of the highest scoring version were considered within round 2.

In the final round, items were ranked in order of importance, higher ranked items were assigned greater point values to enable calculations of total weighted scores and final overall prioritisation. Weighted scores were calculated by summing the points allocated to each item across all participants, with higher weighted scores indicating greater importance for use in clinical practice. Rankings were compared across rounds and professional groups (diagnosing doctors compared to non-diagnosing doctors and physiotherapists) to assess stability and consistency of prioritisation patterns. Visual inspection of ranking patterns was undertaken to assess convergence of expert opinion. All data analysis was performed in excel.

## Results

### Participant characteristics

Of the 114 experts invited, 38 completed round 1 survey (33.3% response rate). Of those not participating, six declined, two surveys were incomplete, and 68 did not respond. Participant characteristics can be seen in Table 1. The majority (81.6%) were orthopaedic surgeons, of whom 71.0% work exclusively in paediatric practice; nearly half (47.4%) reported more than 20 years’ experience working with children and adolescents with Perthes disease. Overall retention rates between rounds two and three were high with 92.1% and 89.5% respectively.

**Table 1:** Participant characteristics

	Round 1 (*n* = 38)	Round 2 (*n* = 35)	Round 3 (*n* = 34)
*Participant profession* *n* *(%)*			
Paediatric orthopaedic surgeon	22 (57.9)	19 (54.3)	19 (55.9)
Orthopaedic surgeon	5 (13.1)	5 (14.3)	5 (14.7)
Paediatric Endocrinologist	3 (7.9)	3 (8.6)	3 (8.8)
Paediatric rehabilitation specialist	1 (2.6)	1 (2.8)	1 (2.9)
Physiotherapist*^	6 (15.9)	6 (17.1)	6 (17.6)
PhD Scientist	1 (2.6)	1 (2.8)	0 (0.0)
*Diagnose condition*			
Yes	33 (86.8)	30 (85.7)	30 (88.2)
*Sex n (%)*			
Female	13 (34.2)	12 (34.3)	11 (32.4)
Male	25 (65.8)	23 (65.7)	23 (67.6)
*Country n (%)*			
Australia	16 (42.1)	16 (45.7)	16 (47.1)
New Zealand	2 (5.3)	0 (0.0)	0 (0.0)
Switzerland	2 (5.3)	2 (5.7)	2 (5.9)
United Kingdom	4 (10.5)	3 (8.6)	3 (8.8)
United States of America	10 (26.3)	10 (28.6)	9 (26.5)
Other^^*	4 (10.5)	4 (11.4)	4 (11.8)
*Years of clinical experience n (%)*			
Less than 5	1 (2.6)	1 (2.9)	0 (0.0)
5–9	4 (10.5)	4 (11.4)	4 (11.8)
10–14	8 (21.1)	6 (17.1)	6 (17.6)
15–19	7 (18.4)	6 (17.1)	6 (17.6)
20+	18 (47.4)	18 (51.4)	18 (52.9)
*Workplace healthcare setting^ n (%)*			
*Hospital*			
Paediatric specific	33 (86.8)	30 (85.7)	29 (85.3)
Non-paediatric specific	3 (7.9)	3 (8.5)	3 (8.8)
Private practice	4 (10.5)	4 (11.4)	4 (11.8)
Other‡	3 (7.9)	3 (8.6)	3 (8.8)

Physiotherapist*^: includes physiotherapists and advanced practice physiotherapists; Other^^*: countries each represented by one panel participant – includes India, Ireland, Spain, and Sweden; Other‡ workplace settings, each represented once: Clinical academic, community health, retired; ^: % add to greater than 100% as some participants have dual appointments

#### Round 1

**Fig. 1:**
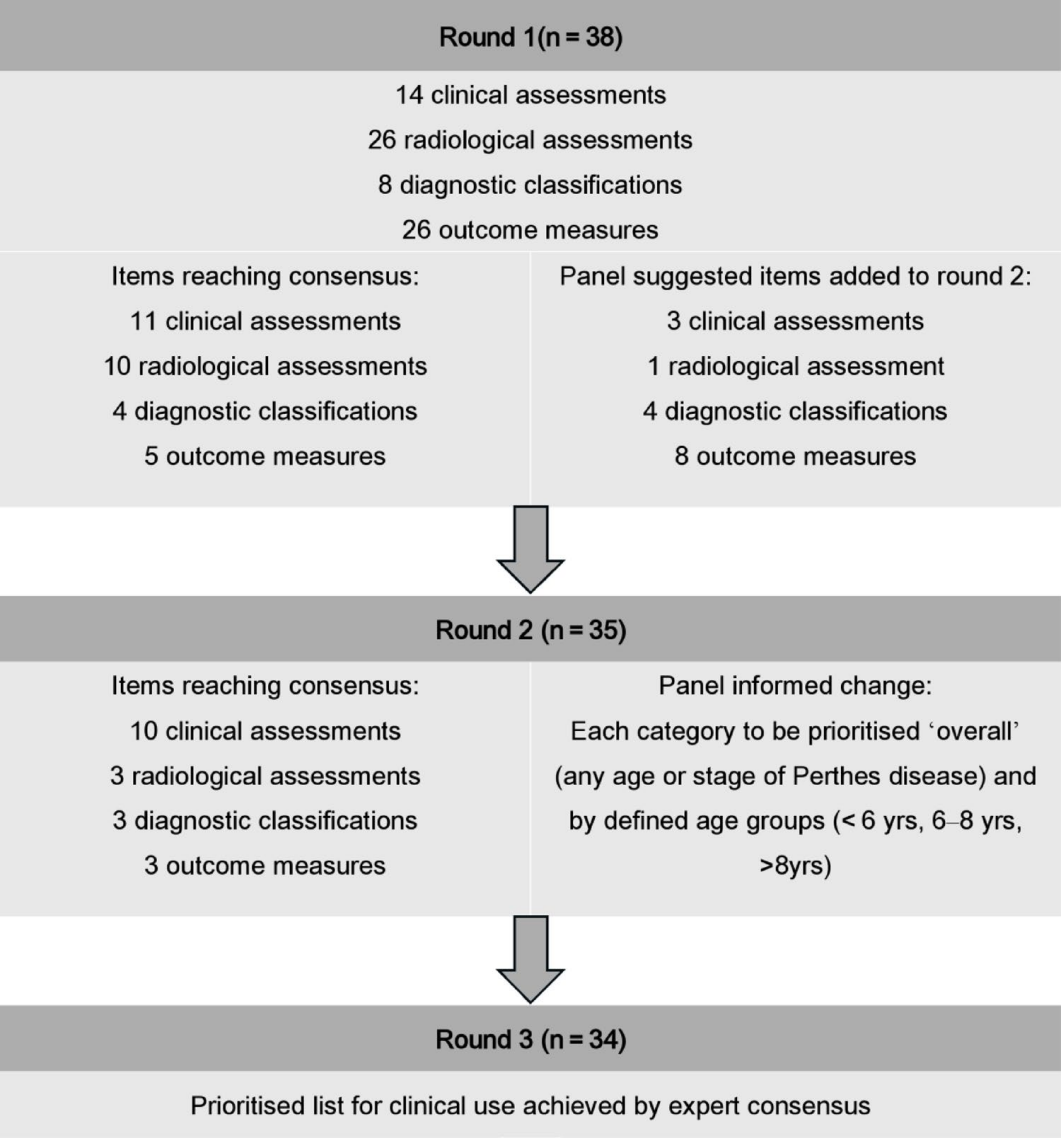
Flow chart showing Delphi process

Consensus was reached on 11 clinical assessments,10 radiological assessments, four diagnostic classifications, and five outcome measures (four clinical, one radiological) (Fig. 1, Online Resource 2). Following review of free-text responses, an additional three clinical assessments, one radiological assessment, four diagnostic classifications, and eight outcome measures were included in round 2.

#### Round 2

Consensus was reached on the importance of 10 clinical assessments, three radiological assessments, three diagnostic classifications, and three outcome measures (two clinical, one radiological) (Fig. 1, Online Resource 2). Differences in agreement were present between health professionals who diagnose and those who do not. Those who diagnose placed greater emphasis on impairment-based assessments and surgical outcomes, whilst non-diagnosing participants, including physiotherapists (diagnosing and non-diagnosing), prioritised functional assessments and pain-based outcome measures as clinically important. Analysis of free-text responses informed the prioritisation of items within each category to be completed as ‘overall’ and by specific age groups (< 6 years, 6–8 years, and > 8 years) in round 3 (Fig. 1).

#### Round 3

Prioritisation of assessments, diagnostic classifications, and outcome measures were achieved (Table 2). Rankings were largely consistent between overall and age-specific analyses across all categories, with similar prioritisation patterns observed between diagnosing and non-diagnosing participants. Passive hip ROM and activity restriction status were ranked first and second, respectively, for clinical use in all groups, with minimal variation in other clinical assessment rankings. For radiological assessments, the only difference occurred for children < 6 years, where hinge abduction x-ray was ranked higher than perfusion MRI. Diagnostic classifications showed no between-group variation. PROMIS child-reported was prioritised as the most important outcome measure across all subgroups, except in children < 6 years, where the parent proxy reported version was prioritised highest, consistent with validated use. See Online Resource 2 and 3 for round 3 weighted score results and age-specific rankings.

**Table 2:** Items that reached consensus and prioritised in order of clinical importance

	Participants who diagnose (*n* = 30) Overall
*Diagnostic classification*	
Modified Waldenström classification	1
Modified Herring (Lateral Pillar) classification	2
Perfusion MRI	3
*Radiological assessments*	
Lateral subluxation of the femoral head	1
MRI: perfusion	2
x-ray: hinge abduction	3

	All participants (*n* = 34) Overall
*Outcome measures*	
PROMIS – Child version	1
Stulberg hip classification	2
PROMIS – Parent version	3
*Clinical assessments*	
Hip ROM – Passive	1
Activity restriction status	2
Limping	3
Frequency of pain medication use	4
Gait	5
Hip ROM – Active	6
Missing days from school/childcare due to pain	7
Adverse surgical outcomes – requires further surgery	8
Leg length discrepancy	9
Trendelenburg	10

*n* number, *yrs* age in years, *MRI* magnetic resonance imaging, *PROMIS* Patient reported outcome measures information system, *ROM* range of motion

## Discussion

To our knowledge, this is the first study to reach consensus and prioritise assessments, diagnostic classifications, and outcome measures for use in clinical practice for children and adolescents with Perthes disease by an expert international panel. The final set of items prioritised within each category provide methods to evaluate each domain of the core outcome set for Perthes disease [19] and can be used in clinical practice for children and adolescents to facilitate standardised outcome reporting, improve communication, and clinical decision making.

The numerous and variable assessments and classifications currently in use presents a challenge for clinicians in determining which are most appropriate to use in specific clinical contexts, highlighting the need to reach consensus in methodological approaches. Variability was seen within this study, with the addition of multiple items within all categories following suggestion by panel members after round 1. Despite this, a consensus driven, consolidated minimum set of methods were identified that is specific to Perthes disease and psychometrically appropriate, enabling more succinct assessment, diagnosis, and evaluation of outcomes for these patients [2, 13, 14, 22, 30]. Standardising methodology for measuring and reporting outcomes is an important step towards enhancing comparability and optimising patient outcomes [2].

The relevance and importance of patient reported outcome measures has been established [29]. Two of the three outcome measures reaching consensus in this study demonstrate patient-centred approach to care. The publicly available National Institute of Health designed PROMIS provides clinicians with insight into the patient perspective on the impact of physical symptoms and functional status on psychological and social functioning [1, 6, 9]. Construct validity of PROMIS has been established for use in Perthes disease, measuring health-related quality of life at different stages of the disease [2]. The design of PROMIS is to be efficient, relevant, and psychometrically competent for paediatric populations of all ages, via self-reporting (children > 8 years) or parent as proxy reporters (children < 8 years or with cognitive impairments). In this study, self-report was prioritised for clinical use overall and in all age groups except children < 6 years.

Implementation of patient reported outcome tools into routine clinical practice may be considered advantageous for patient-centred practice and understanding overall outcomes, whilst a possible challenge for health professionals. A paediatric specific study assessed the inhibitors and facilitators of implementing patient reported outcomes into clinical practice from multiple stakeholder perspectives [23]. A key feature inhibiting implementation was time constraints on treating health professionals, which was also a reported concern by participants of this study. However, in adolescents with idiopathic scoliosis, it has been found that it takes an average of 0.9–1.4 min to complete an individual PROMIS computer adaptive test, which is similar to adult populations, demonstrating low impact upon patients and the healthcare team [27]. Further, clinicians can choose particular banks of questions relevant to the time point of assessment. PROMIS is easily accessible, available in multiple languages, and formats to support individualised delivery at appropriate timepoints.

Perthes disease is ranked among the top ten paediatric musculoskeletal conditions contributing to chronic lower limb pain prioritised for clinical practice guideline development, reflecting considerable concern for patients, families, and health professionals, and highlighting the need to alleviate pain and improve functional outcomes [8, 11]. While specific pain assessments or outcome measures did not reach consensus in the final round of this study, pain was indirectly measured via frequency of pain medication use and days off school due to pain, which were both prioritised in the final round. Five pain specific measures were presented to participants as identified within the literature and an additional measure was suggested after round 1. Fourteen outcome measures presented to participants incorporated a pain measure component of which PROMIS (child and parent-proxy reporter) were the only measures to reach consensus. Therefore, without any specific pain assessments reaching consensus and prioritisation, the implementation of PROMIS into routine clinical practice is important to ensure children and adolescents lower limb pain is assessed, monitored and addressed where needed.

Two out of the three diagnostic classifications reaching consensus within this study were specifically developed for Perthes disease and have undergone modifications since their inception to improve sensitivity, and inter and intra-observer reliability [7, 13–15, 18]. Both modified Herring lateral pillar and modified Waldenström classifications have good to excellent interobserver and intraobserver reliability. However, studies have demonstrated variability from disease stage time and level of expertise of reviewer for both classifications [7, 13, 14, 18]. Each classification has its limitations in clinical practice. For example, modified Herring lateral pillar classification is validated for use in the fragmentation stage of the disease, whilst also supporting the prognostication of disease outcome [13]. Whereas modified Waldentröm classification provides staging of the disease and is useful for informing treatment choices [15]. Perfusion MRI is seen to be more readily used in early stages of the disease, in particular for the older child, and where changes are not visible on radiographs [4]. Perfusion MRI is infrequently used in younger children as they typically have favourable remodelling potential when disease onset occurs early and due to the frequent need of anaesthesia to conduct the examination [4].

Whilst this study included a diverse international panel of experts with extensive experience managing children and adolescents with Perthes disease, and high retention rates across Delphi rounds, limitations should be acknowledged. Orthopaedic surgeons comprised the largest professional group within the panel, reflecting the central role of orthopaedic management in Perthes disease care. The perspectives of other health professionals may have been underrepresented, particularly as access to multidisciplinary care varies across patients and healthcare settings [5]. Whilst greater recruitment of panel members may broaden the perspectives and generalisability of the consensus results, there is no universally accepted sample size for Delphi studies in healthcare, particularly one focussed on a rare condition like Perthes disease [24, 25]. While health professionals from all regions of the world were invited to participate, representation from Asia was limited and no participants from South America were recruited, which may reduce the applicability of our findings across geographical regions where healthcare contexts may differ. Although all multidisciplinary team disciplines were invited, participation was limited to medical professionals and physiotherapists. Differences in agreement between health professionals based on discipline was seen in round 2, whereby physiotherapists (those who diagnose and do not) and non-orthopaedic doctors placed great emphasis on pain scales and functional outcomes, whereas orthopaedic surgeons prioritised radiological and impairment-based assessments. This demonstrates the differences in professional scope and reinforces the importance of a multidisciplinary team approach to managing children with Perthes disease to address the broad impact of the condition. Despite differences, strong agreement was observed between all disciplines when prioritising items in the final round. This Delphi survey was designed to obtain expert consensus; however, the absence of patient and family members on the panel is an important limitation, as the prioritised items may not fully capture the outcomes and assessments most meaningful to children, adolescents, and families affected by Perthes disease.

Paediatric orthopaedic specialists have reached consensus and prioritised assessments, diagnostic classifications, and outcome measures important for clinical use for patients with Perthes disease. These results are an important step toward improving consistency of care and facilitating the development of clinically relevant, consensus driven clinical practice guidelines for health care professionals managing Perthes disease. Health professionals managing children and adolescents with Perthes disease are encouraged to utilise this minimum set of methods that address the domains of the core outcome set for Perthes disease. Implementation of these prioritised measures both in clinical and research practice may provide an improved opportunity to reduce the variances seen in patient management and overall outcomes.

## Supplementary Information

Below is the link to the electronic supplementary material.

Supplementary Material 1

Supplementary Material 2

Supplementary Material 3

## Data Availability

The anonymised data that support the findings of this study are available upon reasonable request from the corresponding author. The data are not publicly available due to privacy and ethical restrictions.
