# Supplementary material for: Prioritisation of assessments, diagnostic classifications, and outcome measures in Perthes disease: a Delphi survey of international health professionals
Source: Arch Orthop Trauma Surg. 2026 Jun 25;146(1):237. doi: 10.1007/s00402-026-06392-3 (PMC13303684; doi:10.1007/s00402-026-06392-3)
Supplement: Supplementary file 2 — Supplementary Material 2 [file 402_2026_6392_MOESM2_ESM.docx]

**Prioritisation of assessments, diagnostic classifications, and outcome measures in Perthes disease: a Delphi survey of international health professionals**

**Achieves of Orthopaedics and Trauma**

**Supplementary Table 2. Diagnostic classifications, radiological assessments, clinical assessments, and outcome measures percentage agreement, weighted scores, and ranking**

|  | | **Round 1**  **n=33** | **Round 2**  **n=30** | **Round 3**  **n=30** | |
| --- | --- | --- | --- | --- | --- |
|  |  | **Agreement (%)** | | **Weighted points score** | **Rank** |
| **Diagnostic classification** | | | | | |
| Bucholz Ogden Classification | | 12.1 | - | - | - |
| Catterall Classification | | 48.5 | - | - | - |
| Herring (Lateral Pillar) Classification | | 90.9 | # | - | - |
| Modified Elizabethtown Classification | | 60.6 | - | - | - |
| Modified Herring (Lateral Pillar) Classification | | 93.9 | 86.7 | 57 | 2 |
| Modified Waldenström Classification | | 90.9 | 76.7 | 71 | 1 |
| Salter-Thompson Classification | | 42.4 | - | - | - |
| Waldenström Classification | | 75.7 | # | - | - |
| Perfusion MRI | | - | 80.0 | 52 | 3 |
| Perfusion Index | | - | 63.3 | - | - |
| Deformity Index | | - | 46.7 | - | - |
| Bone Scan | | - | 16.7 | - | - |
| **Radiological assessment** | | | | | |
| Acetabular angle - Sharp angle | | 27.3 | - | - | - |
| Epiphyseal quotient | | 45.5 | - | - | - |
| Head at Risk signs | | 78.8 | 66.7 | - | - |
| Gage’s sign | | 69.7 | - | - | - |
| Calcification lateral to the epiphysis | | 78.8 | 50.0 | - | - |
| Lateral subluxation of the femoral head | | 97.0 | 100.0 | 80 | 1 |
| Presence of horizontal growth plate | | 60.0 | - | - | - |
| Metaphyseal cystic changes | | 78.8 | 60.0 | - | - |
| Joint Space | | 84.4 | 63.3 | - | - |
| Metaphysis rarefication | | 60.6 | - | - | - |
| MRI: femoral head involvement (%) | | 81.8 | 73.3 | - | - |
| Acetabular index | | 48.5 | - | - | - |
| MRI: morphological | | 78.8 | 53.3 | - | - |
| MRI: perfusion | | 81.8 | 80.0 | 52 | 2 |
| Neck shaft angle | | 57.6 | - | - | - |
| Reimers extrusion index | | 84.8 | 53.3 | - | - |
| Shenton line | | 72.7 | - | - | - |
| Sphericity deviation score | | 72.7 | - | - | - |
| x-ray: femoral head involvement (%) | | 87.9 | 70.0 | - | - |
| Acetabulum angle | | 21.2 | - | - | - |
| Articulo-trochanteric distance | | 57.6 | - | - | - |
| Caput index | | 15.1 | - | - | - |
| Caput-collum-diaphyseal angle | | 24.2 | - | - | - |
| Centre-edge angle (of Wiberg) | | 57.6 | - | - | - |
| Centre-trochanteric angle | | 21.1 | - | - | - |
| Consolidation | | 57.6 | - | - | - |
| x-ray: hinge abduction | | - | 76.7 | 48 | 3 |
| **Clinical assessments** | **Round 1**  **n=38** | | **Round 2**  **n=35** | **Round 3**  **n=33** | |
| Ability to sit crossed legged | 68.4 | | - | - | - |
| Ability to squat | 65.8 | | - | - | - |
| Activity monitor | 63.1 | | - | - | - |
| Activity restriction status | 89.5 | | 100 | 229 | 2 |
| Adverse surgical outcomes: infection | 94.7 | | 71.4 | - | - |
| Adverse surgical outcomes: requires further surgery | 94.7 | | 77.1 | 138 | 8 |
| Gait | 94.7 | | 85.7 | 188 | 5 |
| Hip ROM – active | 92.1 | | 75.0 | 182 | 6 |
| Hip ROM – passive | 97.4 | | 97.1 | 275 | 1 |
| Leg length discrepancy | 97.4 | | 85.7 | 128 | 9 |
| Limping | 94.7 | | 91.4 | 213 | 3 |
| Missing days from school/preschool due to pain | 89.5 | | 80.0 | 174 | 7 |
| Muscle strength test | 81.6 | | 48.6 | - | - |
| Trendelenburg | 89.5 | | 82.8 | 127 | 10 |
| Out of chair test | - | | 25.7 | - | - |
| Frequency of pain medication use | - | | 75.0 | 209 | 4 |
| Wong Baker FACES scale | - | | 48.6 | - | - |
| **Outcome measures** |  | |  |  |  |
| Charnley Score | 5.3 | | - | - | - |
| EQ-5D-5L – Child version | 34.2 | | - | - | - |
| EQ-5D-5L – Parent version | 34.2 | | - | - | - |
| Harris Hip Score | 42.1 | | - | - | - |
| Iowa Hip Score | 26.3 | | - | - | - |
| Modified Harris Hip Score | 42.1 | | - | - | - |
| Nonarthritic Hip Score | 36.8 | | - | - | - |
| Numeric pain scale | 57.9 | | - | - | - |
| OUCHER pain scale | 34.2 | | - | - | - |
| PODCI | 60.5 | | - | - | - |
| PROMIS – Child version | 84.2 | | 77.1 | 83 | 1 |
| PROMIS – Parent version | 86.8 | | 77.1 | 58 | 3 |
| PROMIS–Mobility v2.0 CAT | 81.6 | | 68.6 | - | - |
| PedsQL 4.0 | 65.8 | | - | - | - |
| Ratliff’ clinical evaluation | 13.2 | | - | - | - |
| Short form-12 health survey | 21.1 | | - | - | - |
| Strengths and Difficulties Questionnaire | 21.1 | | - | - | - |
| VAS– Pain – Child reported | 78.9 | | 71.4 | - | - |
| VAS – Pain – Parent reported | 60.5 | | - | - | - |
| WOMAC | 26.2 | | - | - | - |
| Wong-Baker FACES Pain rating scale | 52.6 | | - | - | - |
| Severin classification | 24.2 | | - | - | - |
| Stulberg Hip Classification | 78.8 | | 82.6 | 63 | 2 |
| Tonnis Classification of Hip Osteoarthritis | 33.3 | | - | - | - |
| Modified Stulberg Hip Classification | 72.7 | | - | - | - |
| Mose’s method | 42.4 | | - | - | - |
| COPM | - | | 5.7 | - | - |
| Ellipsoidal index | - | | 11.4 | - | - |
| Sphericity deviation score | - | | 45.7 | - | - |
| MRI - cartilage physis | - | | 34.3 | - | - |
| MRI- bone status | - | | 40.0 | - | - |
| Missing days from school | - | | 65.7 | - | - |
| 6-minute walk test | - | | 28.6 | - | - |
| Child Health Utility instrument (CHU-9D) | - | | 11.4 | - | - |

n: number; MRI: magnetic resonance imaging; ROM: range of motion; PODCI: Paediatric Outcome Data Collection Instrument; PROMIS: Patient reported outcome measures information system; VAS: Visual analogue scale; WOMAC: Western Ontario and McMaster Universities Osteoarthritis Index; COPM: Canadian Occupational Performance Measure; EQ-5D-5L: EquroQual-5 Dimension 5 questions; PedsQL4.0: Pediatric Quality of Life Inventory; #: Where the original and modified versions of a tool scored >75% in round 1, the results of the highest scoring version are presented for Round 2.
