# Supplementary material for: Prioritisation of assessments, diagnostic classifications, and outcome measures in Perthes disease: a Delphi survey of international health professionals
Source: Arch Orthop Trauma Surg. 2026 Jun 25;146(1):237. doi: 10.1007/s00402-026-06392-3 (PMC13303684; doi:10.1007/s00402-026-06392-3)
Supplement: Supplementary file 3 — Supplementary Material 3 [file 402_2026_6392_MOESM3_ESM.docx]

**Prioritisation of assessments, diagnostic classifications, and outcome measures in Perthes disease: a Delphi survey of international health professionals**

**Achieves of Orthopaedics and Trauma**

**Supplementary Table 3. Items in each category that reached consensus prioritised in order of clinical importance overall, and by specific age groups**

|  | Diagnosing participants (n=30)  **OVERALL** | Diagnosing participants (n=30)  **<6yr** | Diagnosing participants (n=30)  **6–8 yr** | Diagnosing participants (n=30)  **>8yr** |
| --- | --- | --- | --- | --- |
| **Diagnostic classifications** | | | | |
| Modified Herring (Lateral Pillar) Classification | 2 | 2 | 2 | 2 |
| Modified Waldenström Classification | 1 | 1 | 1 | 1 |
| Perfusion MRI | 3 | 3 | 3 | 3 |
| **Radiological assessments** | | | | |
| Lateral subluxation of the femoral head | 1 | 1 | 1 | 1 |
| MRI: perfusion | 2 | 3 | 2 | 2 |
| x-ray: hinge abduction | 3 | 2 | 3 | 3 |
|  | All participants (n=34)  **OVERALL** | All participants (n=34)  **<6yr** | All participants (n=34)  **6–8 yr** | All participants (n=34)  **>8yr** |
| **Outcome measures** | | | | |
| PROMIS – Child version | 1 | 3 | 1 | 1 |
| Stulberg Hip Classification | 2 | 2 | 2 | 2 |
| PROMIS – Parent version | 3 | 1 | 3 | 3 |
| **Clinical assessments** | | | | |
| Hip ROM – Passive | 1 | 1 | 1 | 1 |
| Activity restriction status | 2 | 2 | 2 | 2 |
| Limping | 3 | 3 | 4 | 3 |
| Frequency of pain medication use | 4 | 4 | 3 | 4 |
| Gait | 5 | 5 | 7 | 5 |
| Hip ROM – Active | 6 | 6 | 5 | 7 |
| Missing days from school/childcare due to pain | 7 | 7 | 6 | 6 |
| Adverse surgical outcomes – requires further surgery | 8 | 10 | 8 | 8 |
| Leg length discrepancy | 9 | 8 | 9 | 9 |
| Trendelenburg | 10 | 9 | 10 | 10 |

PROMIS: Patient reported outcome measures information system; ROM: range of motion: number; yrs: age in years; MRI: magnetic resonance imaging
